# Structural, Biochemical and Genetic Characterization of Dissimilatory ATP Sulfurylase from *Allochromatium vinosum*

**DOI:** 10.1371/journal.pone.0074707

**Published:** 2013-09-20

**Authors:** Kristian Parey, Ulrike Demmer, Eberhard Warkentin, Astrid Wynen, Ulrich Ermler, Christiane Dahl

**Affiliations:** 1 Max-Planck-Institut für Biophysik, Frankfurt, Germany; 2 Institut für Biophysik und Physikalische Biochemie, Universität Regensburg, Regensburg, Germany; 3 Institut für Mikrobiologie & Biotechnologie, Rheinische Friedrich-Wilhelms-Universität Bonn, Bonn, Germany; Universidade Nova de Lisboa, Portugal

## Abstract

ATP sulfurylase (ATPS) catalyzes a key reaction in the global sulfur cycle by reversibly converting inorganic sulfate (SO_4_^2−^) with ATP to adenosine 5′-phosphosulfate (APS) and pyrophosphate (PP_i_). In this work we report on the *sat* encoded dissimilatory ATP sulfurylase from the sulfur-oxidizing purple sulfur bacterium *Allochromatium vinosum*. In this organism, the *sat* gene is located in one operon and co-transcribed with the *aprMBA* genes for membrane-bound APS reductase. Like APS reductase, Sat is dispensible for growth on reduced sulfur compounds due to the presence of an alternate, so far unidentified sulfite-oxidizing pathway in *A. vinosum*. Sulfate assimilation also proceeds independently of Sat by a separate pathway involving a *cysDN*-encoded assimilatory ATP sulfurylase. We produced the purple bacterial *sat*-encoded ATP sulfurylase as a recombinant protein in *E. coli*, determined crucial kinetic parameters and obtained a crystal structure in an open state with a ligand-free active site. By comparison with several known structures of the ATPS-APS complex in the closed state a scenario about substrate-induced conformational changes was worked out. Despite different kinetic properties ATPS involved in sulfur-oxidizing and sulfate-reducing processes are not distinguishable on a structural level presumably due to the interference between functional and evolutionary processes.

## Introduction

Sulfur compounds are used by a huge variety of organisms for the biosynthesis of sulfur-containing amino acids, cofactors and metabolites [1] and for energy conservation serving either as electron donors or acceptors [2]. Sulfur occurs in the biosphere in oxidation states –II to +VI, mostly in form of hydrogen sulfide, elemental sulfur and sulfate. The dissimilatory sulfur-oxidizing and sulfate-reducing as well as the ubiquitous assimilatory sulfate-reducing processes share several key enzymes, namely ATP sulfurylase (ATPS), adenosine-5-phosphosulfate reductase (APSR) and sulfite reductase (Sir) [2], [3].

In the sulfur-oxidizing pathway ATP sulfurylase (adenylsulfurylase/ATP:sulfate adenylyltransferase; E.C. 2.7.7.4) catalyzes the final reaction of the oxidation of reduced sulfur compounds by reversibly transferring pyrophosphate (PP_i_) onto adenosine-5′-phosphosulfate (APS) to form inorganic sulfate (SO_4_^2−^) and ATP [4], [5], [6]: MgPP_i_+APS 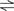 MgATP+SO_4_^2−^. In addition, the ATPS reaction provides a major route for recycling PP_i_ produced by biosynthetic reactions.

In the sulfate-reducing pathway ATPS catalyzes the adenylation of SO_4_^2−^ with ATP to APS and PP_i_. APS is used as the activated form of sulfate in the dissimilatory process and in the assimilatory process of plants, algae and most bacteria whereas phosphoadenosine-5′-phosphosulfate (PAPS) generated from APS by phosphorylation exerts this function in the assimilatory pathway of some organisms such as fungi and some bacteria including most cyanobacteria [2], [7], [8]. In addition, PAPS serves as the sulfuryl donor for the formation of sulfate esters by sulfotransferases.

Except for a number of bacteria containing a ATPS consisting of four heterodimers (CysDN) for assimilatory sulfate reduction [8], [9], a common fold for catalyzing the ATPS reaction occurs for the remaining organisms. In yeast [10], filamentous fungi [11], and bacterial species the assimilatory sulfate-reducing ATPS have been described as homohexamers [12] and in plants as homotetramers [13] of 41–69 kDa-subunits. In some enzymes an APS kinase domain is C-terminally fused to ATPS [8], [14]. The APS kinase domain can be enzymatically active as in *Aquifex aeolicus* [15], possess a regulatory function by binding the allosteric inhibitor PAPS as in filamentous fungi [11] or have no defined function as in yeast [10]. In higher eukaryotes, like in mammalian species [16] or in metazoan organisms, APS kinase is N-terminally fused to ATPS. Sulfate-reducing dissimilatory ATPS is present as a homotrimer with one Zn ion bound to each ATPS subunit [17]. Dissimilatory sulfur-oxidizing ATPS was found to exist as a homodimer [12].

The dissimilatory oxidation of reduced inorganic sulfur compounds is linked to energy transformations via photosynthesis or respiratory processes. Sulfur oxidizers are found among the *Archaea* and the *Bacteria* and comprise photo- and chemolithotrophs. Dissimilatory sulfur oxidation in *Eukarya* is mediated by lithotrophic bacterial endosymbionts. In the phototrophic purple sulfur bacterium *Allochromatium vinosum* two types of ATPSs where identified on the basis of DNA sequence analysis and by inspection of the complete genome sequence [18]. The genes *cysDN* (Alvin_2448 and Alvin_2449) encode for ATPS in an unusual sulfate assimilation pathway [19] and the gene *sat* (Alvin_1118) for an ATPS involved in the dissimilatory sulfur oxidation pathway, respectively. We have overproduced ATPS of *A. vinosum*, characterized the enzyme kinetically, determined its structure of the *A. vinosum* enzyme in an open state and analyzed the conformational rearrangement upon APS and PP_i_ binding.

## Results and Discussion

### The *sat-aprMBA* Gene Locus in *A. vinosum* and other Phototrophic Members of the Family Chromatiaceae

In *A. vinosum* the *sat* gene encoding ATP sulfurylase (Alvin_1118) is located immediately upstream of the *aprMBA* genes encoding membrane-bound APS reductase (Alvin_1119–1121) [3], [18]. AprM is predicted to contain five transmembrane helices with no sequence similarity to any currently known conserved domain or cofactor binding site in the databases. An essential function of AprM as a membrane anchor that allows spatial and functional association of this type of oxidative APS reductase with the membrane has been postulated and it has been suggested that AprM serves as an entry point into the membrane for the electrons released during formation of APS from sulfite and AMP [20]. In the currently available complete genome sequences of phototrophic members of the family Chromatiaceae, the same gene arrangement is present in *Thiorhodovibrio* sp. 970. In *Thiocapsa marina* 5811 (DSM 5653^T^), *Thiorhodococcus drewsii* AZ1 (DSM 15006^T^) and *Thioflaviococcus. mobilis* DSM 8321^T^ *sat* and *aprMBA* are not linked on the chromosome. The occurrence of *aprMBA* has also been reported for *Thiococcus. pfennigii* 4520 [21] while *Thiocystis violacescens* DSM 198^T^ encodes Sat, AprBA and QmoABC, each in separate loci. The QmoABC complex was first identified in the dissimilatory sulfate reducing bacterium *Desulfovibrio desulfuricans* [22]. The complex consists of one membrane (QmoC) and two cytoplasmic subunits (QmoAB). The two QmoC hemes *b* are reduced by quinols and experimental evidence strongly indicates that the Qmo complex participates in electron flow between the quinone pool and the cytoplasm, i.e. that it acts as the electron-donating unit for APS reductase in sulfate reducers [23], [24]. The *qmoABC* genes are not only present in sulfate-reducing prokaryotes [23] but occur also in many chemotrophic sulfur-oxdizing bacteria as well as in green sulfur bacteria [24], [25] and in one further purple sulfur bacterium (*Thiodictyon* sp. Cad16 [21]). In sulfur oxidizers, QmoABC is thought to act as electron acceptor for the electrons released during formation of APS and would thus have a function analogous to that of AprM It should be noted that purple sulfur bacteria do not use a single mechanism to oxidize sulfite which is apparent from our former findings that APS reductase is not essential for sulfite oxidation in *Allochromatium vinosum* [26], [27] and also from the observation that the complete genome of *Marichromatium purpuratum* 984 (DSM 1591^T^) neither contains *sat* nor *aprBA* genes and *apr* genes are also not present in *Isochromatium* [20].

### Insertional Inactivation of the *sat* Gene in *A. vinosum*

Experimental evidence for the *sat-aprMBA* genes forming a transcriptional unit was obtained by introducing a kanamycin-Ω cassette into the *sat* gene resulting not only in a nearly complete loss of ATP sulfurylase activity (in extracts of cells grown photolithoautotrophically on sulfide for the wildtype 0.3 U (mg protein) ^−1^ and for the mutant 0.001 U (mg protein) ^−1^; activities were determined by using the assay in the APS synthesis direction as outlined in the “Material and methods” section) but also APS reductase activity in crude extracts of the purple sulfur bacterium. The *A. vinosum sat*::Ωkm mutant strain was still able to grow photolithoautotrophically on sulfide and also photoorganoheterotrophically on sulfate as the sole sulfur source. These findings imply that the *sat*-encoded enzyme is neither essential for sulfate assimilation nor for dissimilatory sulfur oxidation in this organism. These results are corroborated by an earlier report that the ability to indirectly oxidize sulfite via APS is not required for *A. vinosum* [26] and that a dedicated pathway exists for sulfate assimilation [19].

### Kinetic Characterization of Recombinant *A. vinosum* ATP sulfurylase

**Figure 1:**
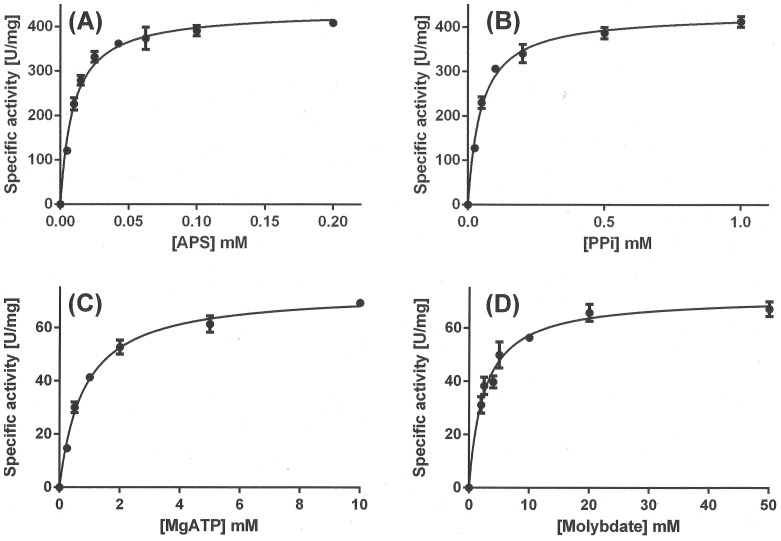
Kinetics of recombinant ATP sulfurylase from *A. vinosum*. [A] *v versus* [APS] in the ATP synthesis reaction at 1 mM pyrophosphate. [B] *v versus* [PP_i_] at 0.2 mM APS. [C] *v versus* [MgATP] in the molybdolysis reaction at 50 mM MoO_4_^2−^. [D] *v versus* [molybdate] in the molybdolysis reaction at 10 mM MgATP. Each data point is the mean ± standard deviation of three assays on the same batch of protein but in some cases the error bars are too small to be seen. The solid lines through the data points are the fits to the Michaelis-Menten equation using non-linear regression as described in Materials and Methods.

The recombinant *A. vinosum* ATP sulfurylase produced in *E. coli* was tested for enzymatic activity *in vitro* and some crucial kinetic parameters were determined (Fig. 1). The enzyme displayed hyperbolic *v versus* plots (Fig. 1) and normal (linear) reciprocal plots. Our measurements showed a *V*_max_ in the direction of ATP synthesis of 433±18 U (mg protein) ^−1^ at 30°C, pH 8.0 and saturating substrate concentrations (1 mM PP_i_ and 0.2 mM APS). The observed specific activity is in the range of that reported for the enzyme from “*Candidatus* Endoriftia Persephone” [12], the sulfur oxidizing symbiont of *Riftia pachyptila*. K_M_ values for APS and pyrophosphate were determined to be 9.5 µM and 47.6 µM, respectively. In the molybdolysis assay *V*_max_ was 72.3±4.1 U (mg protein) ^−1^, K_M_ values for ATP and molybdate were determined to be 0.8 mM for ATP and 2.5 mM for molybdate. A much higher specific activity in the direction of ATP production from APS and PP_i_ than in the molybdolysis assay has also been observed for “*Candidatus* E. persephone” ATPS while this ratio is generally smaller in assimilatory ATPS indicating a special adaption of the enzymes from sulfur oxidizers for working effectively in the ATP synthesis direction.

### Structure Overview

The X-ray structure of ATPS from *A. vinosum* overproduced in *E. coli* was refined to R/R_free_ factors of 17.9%/20.2%. The diffraction data were incompletely collected to 1.6 Å (38% completeness in the highest resolution shell), but at 1.8 Å resolution the local completeness is 82.3% and the accumulated completeness is 94.7% (see Table 1 for further data statistics).

**Table 1:** Data collection and refinement statistics.

Data collection	
Detector type	ADSC Quantum 4 ccd
X-ray wavelength (Å)	0.9393
Space group	P2_1_
Unit cell parameters (Å)(°)	a = 73.3, b = 97.0, c = 73.5117.6
Resolution range (Å)	1.6–19.8 (1.66–1.60)
No. of observed reflections	98813 (4546)
Completeness (%)	83.4 (38.6)
Multiplicity	2.2 (1.4)
I/σ(I)	15.8 (2.1)
R_merge_ (%)	6.0 (29.9)
**Model refinement**	
Resolution range (Å)	1.6–19.8 (1.65–1.60)
R*_work_*/R*_free_* (%)	17.9/20.2 (31.8/36.6)
Overall B factor (Å^2^)	
Protein	23.7
Water	30.3
R.m.s deviations from ideal geometry	
Bond lengths (Å)	0.017
Bond angles (°)	1.65
Ramachandran plot	
Most favorable (%)	99.0
Allowed (%)	0.75
Generously allowed (%)	0.25
Disallowed (%)	0.0
PDB code	4DNX

Due to the low completeness of the data in the highest resolution shell, the effective resolution is lower than 1.6 Å.

**Figure 2:**
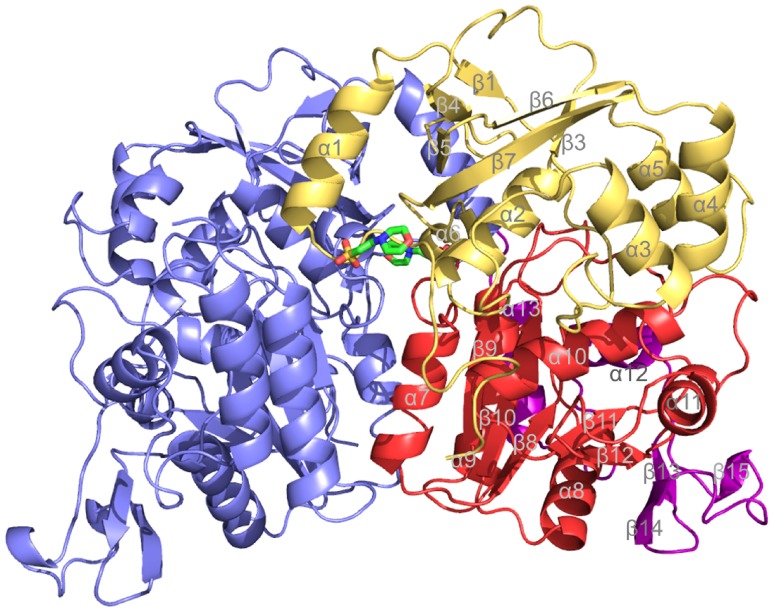
Structure of ATPS from *A. vinosum* organized as a homodimer with a size of 70 × 50 × 50 Å^3^. The subunits are drawn in blue and red/orange; domains I, II and III of one subunit in orange, red and dark red. 2N-Morpholino-ethansulfonic acid (MES) is shown as stick model.

However, the inclusion of the incomplete higher-resolution data in the refinement improved the quality of the electron density maps and was essential to our structure analysis. The asymmetric unit contains one homodimer of ATPS with rms differences between the monomers being 1.4 Å. No significant differences were detectable between them except for a rigid-body movement of the C-terminal relative to the other domains in the range of 4 Å and of segment 233∶239 which contacts the C-terminal domain. As described previously for ATPS structures from other organisms [28] each subunit of *A. vinosum* ATPS is subdivided into three domains (Fig. 2). The N-terminal domain I (residues 1–172) is basically built up of a β barrel with five antiparallel strands surrounded by α-helices, the catalytic domain II (residues 173–331) of a typical Rossmann-like α/β structure and the small C-terminal domain III (residues 332–396) of a small three-strand β-sheet and two α-helices. As mentioned, ATPS structures of various assimilatory sulfate reducing organisms such as fungi, yeast and *Aquifex aeolicus* [14], [28], [29] contain a fourth APS kinase-like domain. The structure of ATPS of *A. vinosum* was determined without any ligand in the active site. Two MES (2N-morpholino-ethansulfonic acid) molecules (present in the crystallization solution) could be, however, identified close to the monomer-monomer interface far away from the active site (Fig. 2). They are primarily linked to the polypeptide by hydrogen bonds between the oxygen of its morpholino ring and a guanidino group of Arg13 and between two sulfate oxygens and the amide nitrogen of Asp17.

### The Substrate Binding Process

**Figure 3:**
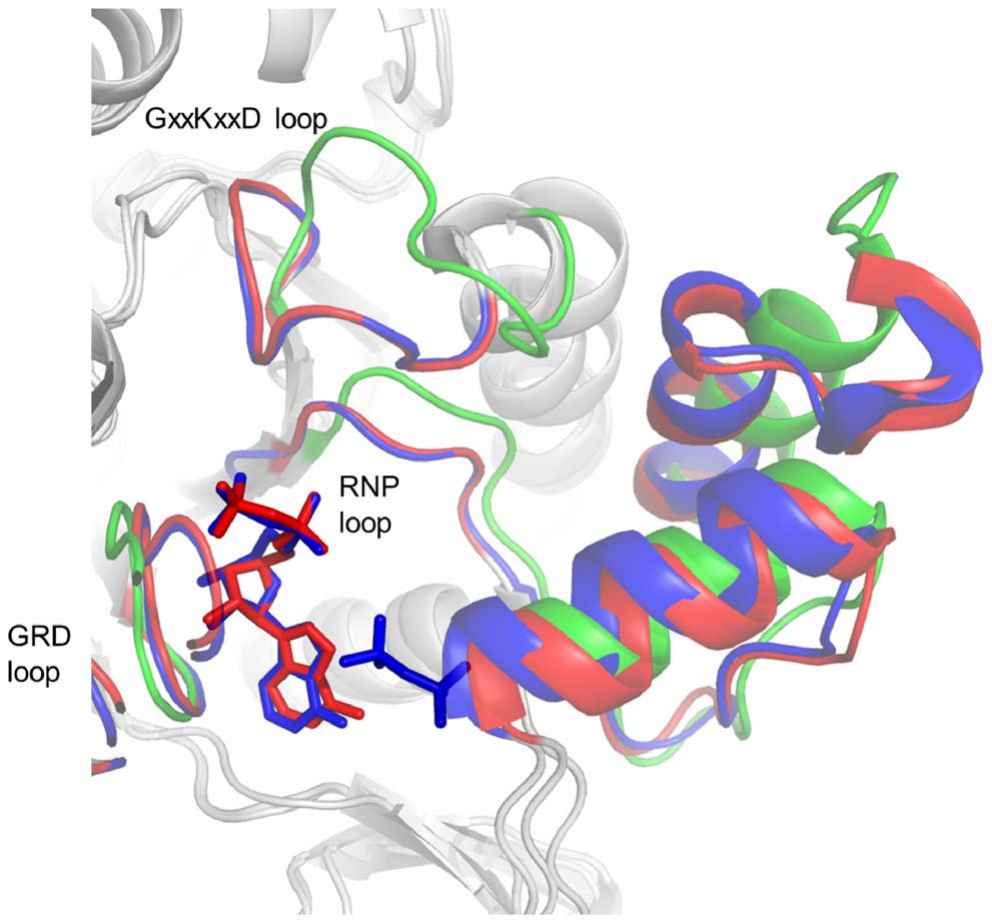
The substrate binding site. The structures of the ligand-free ATPS of *A. vinosum* (red), the ATPS-APS of *P. chrysogenum* (green) and the ATPS-APS-PP_i_ of *S. cerevisiae* (blue) are superimposed thereby focusing on the pronounced loop segments that are subjected to a conformational change upon substrate binding. As a result, the substrate binding groove is shrunk from an open form characterized in the *A. vinosum* and “*Candidatus* E. persephone” ATPS structures to a more closed form found in the structures of *S. cerevisiae, P. chrysogenum, Aquifex aeolicus* and *T. thermophilus* ATPS in complex with the substrates (or substrate analogues).

The substrate binding and active sites are positioned in a deep groove above the C-terminal ends of the central β-sheet of domain II; its walls are composed of the loops following strands 8 (195∶199), 9 (224∶230), 10 (260∶266), 11 (291∶295), and 12 (326∶329) as well as of the N-terminal side of helix 12 (361∶370) (Fig. 3). The characteristic RNP (^199^QXRN^202^) and GRD (^295^GRD^297^) loops follow the straddled strands 8 (195∶199) and 11 (291∶295). ATPS has been structurally analyzed from several organisms in various active site ligation states. In the *A. vinosum* ATPS structure the active site is only occupied by water molecules; more than ten of them are visible in the electron density map. Substrate-free *Saccharomyces cerevisiae*, “*Candidatus* E. persephone” and human ATPS structures contained phosphate, sulfate or chloride molecules attracted by patches of positively charged residues of the active site groove (Fig. S2) [28], [30], [31]. The *S. cerevisiae*, *Penicillium chrysogenum*, *Aquifex aeolicus* and *Thermus thermophilus* ATPS structures were determined in complex with APS [14], [28], [29], [32] and yeast ATPS with APS (or ATP analogues), pyrophosphate and chromate [33]. On this structural basis a scenario for the induced-fit process as a result of substrate binding is proposed. The catalytic process starts by binding APS into the empty active site groove best expressed by the *A. vinosum* ATPS structure present in an open form (Fig. 3). Docking experiments using the program AutoDock [34] revealed that the substrate APS also binds with a significant affinity to the enzyme conformation of the substrate-free state (Fig. S1). Accordingly, APS is attached along the C-terminal end of the central β-sheet of domain II in an L-shaped conformation guided by the positive surface potential.

Initial APS binding activates an induced-fit process resulting in a net shrinkage of the groove from an open to a more closed form observable when comparing ligand-free and APS bound ATPS structures [30], [28] (Fig. 3). The interactions between the polypeptide and APS are significantly increased during the induced-fit process which might be necessary to fix APS in the found compressed L-shaped conformation. Attracted by APS, the RNP and GRD loops move 2–3 Å and 1–2 Å, respectively, to form hydrogen bonds between the sulfate oxygens of APS and Gln199-N_ε2_H, Arg201-N_η_H_2_ and Ala299-NH, between the α-phosphate oxygens and Thr200-Oγ_1_H, Arg201-NH, Asn202-NH and Asn202-N_δ2_H_2_ and between the adenosine ribose hydroxo groups and Gly295-NH, Arg296-O and His298-N_δ1_. The induced-fit movement is propagated to segments contacting the RNP and GRD loops including the loops that follows strands 9 (224∶230) and 10 (260∶266), helix 9 (240∶254) and the preceding ^231^GxxKxxD^237^ loop, helix 13 (381∶393) and finally to the entire C-terminal domain (Fig. 3).

**Figure 4:**
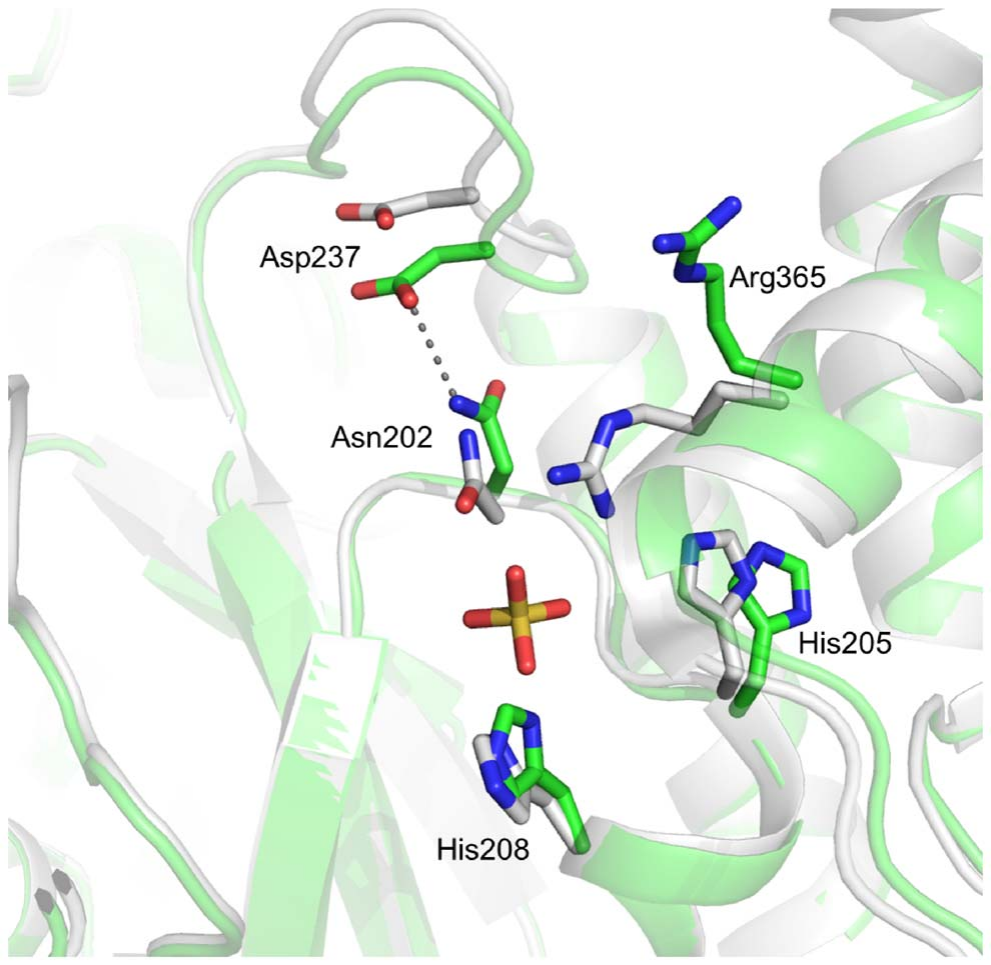
Pyrophosphate binding regions in *A.vinosum* (green) and of “*Candidatus* E. persephone” (grey) ATPS. Phosphate binding found in “*Candidatus* E. persephone” ATPS induces conformational changes of His208 of the ^205^HXXH^208^ motif, Asn202 that influences the conformation of the “GxxKxxD” loop and of Arg365 that moves into the PP_i_ binding site.

The conserved GxxKxxD loop after strand 9 (224∶230) is in an “up” position and highly mobile in the open *A. vinosum* and “*Candidatus* E. persephone” ATPS. Its mobility is expressed by its increased temperature factor (32.4 Å^2^; overall 14.2 Å^2^) and high conformational flexibility of up to 2.5 Å calculated between the two open ATPS structures (Fig. 4). Upon APS binding this loop is shifted 4–9 Å down towards the groove and forms together with the adjacent GRD loop above the phosphosulfate binding site a shield that is absent in the open form (Fig. 3). The up-to-down movement of the GxxKxxD loop is a consequence of the APS induced conformational change of the RNP loop allowing the formation of a hydrogen bond interaction between Asp237 and Arg201 and between Ile238, a solvent molecule and Arg201 and Asn202. Thus, the GxxKxxD loop indirectly participates in APS binding as already recognized by analyzing the “*Candidatus* E. persephone” ATPS structure [30]. In the substrate-free yeast ATPS structure the described close/open transition is largely prevented presumably because a sulfate, bound instead of the sulfate group of APS, is sandwiched between Gln199 and Ala299 and thus fixes a more closed form.

The open form of the C-terminal domain of ATPS of *A. vinosum* interferes with the pyrophosphate binding site which is created upon APS binding in the closed form in front of the N-terminal side of helix 12 (361∶370) [28]. This finding can be correlated with kinetic data that indicated PP_i_ binding only in the presence of APS [35]. (ATPS of *T. thermophilus* is an exception as it contains a more compact active site and would interfere with PP_i_ in the APS bound state of yeast ATPS) [14], [28], [29], [32]. In the yeast ATPS-APS-PP_i_ structure the distance between the substrates is too long for a direct nucleophilic attack suggesting a conformational change of the C-terminal domain (or parts of it) together with PP_i_ prior to the reaction. In the open “*Candidatus* E. persephone” ATPS, the N-terminal region of helix 12 (361∶370) might be biased by two sulfate molecules. On the other hand one of them sits in van der Waals contact to the α-phosphate of APS positioned according to the superimposed yeast and *Aquifex aeolicus* ATPS which were both structurally characterized in complex with APS. This sulfate might occupy the approximate position of the β-phosphate of PP_i_ which performs the nucleophilic attack onto APS forming ATP and sulfate (Fig. 4). (Fig. 4). It interacts with the invariant residues Asn202, His205, His208 and Arg365 that are also involved in pyrophosphate binding and transition state stabilization as shown for mammalian ATPS [36], [37]. A comparison between *“Candidatus* E. persephone” and *A. vinosum* ATPS revealed substantial conformational differences of three of these residues which reflect to a certain extent the induced-fit process upon pyrophosphate binding. His205 is hydrogen-bonded in the *A. vinosum* ATPS with Ser379-Oγ1H and via solvent molecules with Tyr391-OηH and swings towards the active site upon sulfate and presumably also upon APS/PPi binding (Fig. 4). Likewise, Asn202 is hydrogen-bonded with Asp237 in the *A. vinosum* ATPS structure and moves towards the substrates upon their binding. The guanidinium group of Arg365 (part of the conserved ^360^SGTxxR^365^ motif) present in two conformations dominantly points towards Glu366 in *A. vinosum* ATPS and swings more than 6 Å towards the sulfate in the “*Candidatus* E. persephone” ATPS structure (Fig. 4).

### Structural and Sequence Comparisons within the ATP Sulfurylase Family

**Figure 5:**
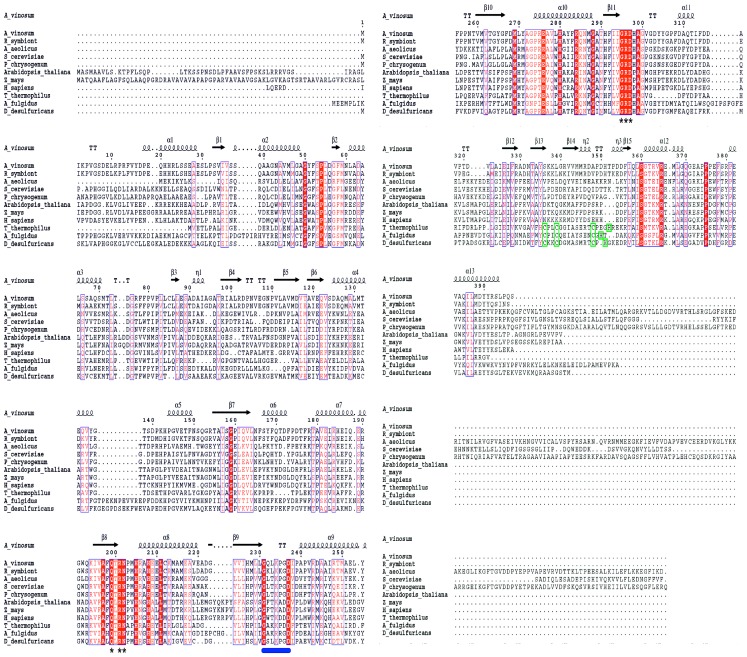
Sequence alignment of ATP sulfurylases from sulfur oxidizing and sulfate assimilating and dissimilating organisms. Residues of the highly conserved RNP and GRD motif and the mobile loop are indicated by asterisks and a blue stripe, respectively. The three cysteines and one histidine of the conserved zinc-binding motif are marked with red boxes. The secondary structure symbols are illustrated according the ATPS structure of *A. vinosum*. The alignment figure was made using the programs ClustalX [50] and ESPript [51]. Species: *Allochromatium vinosum* DSM 180^T^ (A_vinosum, Alvin_1117, ADC62057), “*Candidatus* Endoriftia Persephone” (R_symbiont, PDB: 1JHD_A), *Aquifex aeolicus* VF5 (A_aeolicus, PDB: 2GKS_A), *Saccharomyces cerevisiae* (S_cerevisiae, Met3p, AAU09752), *Penicillium chrysogenum* (P_chrysogenum, MET3_PENCH, Q12650), *Arabidopsis thaliana* (Arabidopsis_thaliana, AAA21570), *Zea mays* (Z_mays, NP_001104877), *Homo sapiens* (H_sapiens, PDB: 2QJF_A), *Thermus thermophilus* (T_thermophilus, YP_004282.1), *Archaeoglobus fulgidus* (A_fulgidus, SAT_ARCFU, SP: O28606.2), *Desulfovibrio desulfuricans* ATCC 27774 (D_desulfuricans, YP_002479044.1).

Sequence comparison studies revealed a high degree of similarity among the ATPS family members (Fig. 5), in particular, for the catalytic domain II which is confirmed by the X-ray structures. The rms deviation between *A. vinosum*, “*Candidatus* E. persephone” [30], yeast [28], fungus [14], human [31], *T. thermophilus* [32], and *Aquifex aeolicus* ATPS [29] ranges between 0.8–2.9% (more than 87% of C^α^ positions used) [38]. Considering that the structures are determined in different ligation states that are accompanied by the described large-scale conformational changes the calculated rms values are strongly overestimated. Equivalent enzymatic states would result in rms deviations below 1 Å for residues of domain II, in particular, for those regions involved in the catalytic reaction.

ATPS functions in three different metabolic contexts. In the widely distributed assimilatory sulfate reduction pathway sulfur is recruited for amino acid and cofactor biosynthesis whereas in the dissimilatory sulfate reduction and sulfide oxidation pathways a limited number of microorganisms consume sulfate and reduced sulfur compounds, respectively, in large amounts because these compounds act as electron donor/acceptors in energy conversion processes. A careful sequence and structural analysis indicates that the RNP and GRD loops and other crucial residues of the groove are strictly conserved except for the six residues longer GRD loop in some assimilatory ATPS that, however, does not directly participate in phosphosulfate binding (Fig. 5). Nevertheless, kinetic data suggest distinct substrate binding and catalysis: APS and PP_i_ synthesis is favoured in sulfate-reducing ATP sulfurylases while the enzymes from sulfur oxidizers appear more adapted to catalyzing ATP and SO_4_^2−^ synthesis [15]. The structural basis for these differences is, however, intricate. Most attractive locations for differences include the irregular region preceding helix 12 (361∶370) of domain III and the GxxKxxD region, both being only moderately conserved (Figs. 2+3). Minor perturbations of their conformations modify the size of the APS and PP_i_ binding sites and the dynamics of the induced-fit process.

Besides the described common factors concerning substrate binding ATPS also reveals differences that more indirectly influence the active site groove but may fine-tune the biochemical reaction. First, sulfur-oxidizing ATPS are present as homodimers, dissimilatory sulfate-reducing ATPS as homotrimers and assimilatory sulfate-reducing ATPS frequently as homotetramers or -hexamers implicating a specific rigidification and stabilization of regions of the active-site groove. First, in *A. vinosum* ATPS the GxxKxxD loop is in direct contact to the partner monomer. Second, assimilatory ATP sulfurylases frequently contain a fourth APS-kinase like domain [28], [14], [29] that represents an essential component of the stable hexamer and influences, in parallel, the ATPS reaction by contacting helix 12 (361∶370) involved in PP_i_ binding. Third, plant ATPS (see Fig. 5 *Arabidopsis thaliana* and *Zea mays* domain I) possesses a more than 50 amino acids longer N-terminal arm that might be involved in oligomeric interactions. Fourth, domain III of ATPS from dissimilatory sulfate reducing microbes and from various sulfur-oxidizing and assimilatory sulfate-reducing organisms contains a characteristic zinc-binding site [32], [17]. The zinc ion is tetrahedrally coordinated by three cysteines and one histidine and is centrally positioned in the solvent-exposed segment that links strand 12 (326∶329) and helix 12 (361∶370) involved in adenine and PP_i_ binding, respectively. The zinc-binding motif preferably found in ATPS of thermophilic organisms appears to be important for active site stabilization when the lack of a APS kinase domain does not allow stabilization by homohexamer formation.

The virtually identical substrate binding site of ATPS - independent of their metabolic functions – arises from its origin very early in evolution prior to the divergence of the three domains of life and from the complex chemical reaction that did not allow substantial variations. Separable classes of ATPS according to their metabolic function are not definable despite the described structural differences and distinguishable kinetic data for the forward and backward reaction. Obviously, subtle adjustments are superimposed by the normal phylogeny of the organisms including the adaptation to specific environmental conditions. One apparent example represents the characteristic zinc-binding motif preferably found in thermophilic microorganisms (Fig. 5). However, a more profound sequence analysis that integrates gene duplication and lateral gene transfer events and a broader functional characterization of individual ATPS is required to explain observations as for example that *Aquifex aeolicus* ATPS is more related to *S. cere*v*isiae* and *P. chrysogenum* ATPS than to most bacterial ATPS or that the plant enzyme is more similar to human ATPS than to the respective protein from green algae [8].

## Materials and Methods

### Construction of *sat*-deficient *Allochromatium vinosum*

A clone carrying a 3-kb *Bgl*II restriction fragment with the complete *sat* gene (pAW307) was isolated from a library of 2.5- to 3.5-kb *Bgl*II restriction fragments of total *A. vinosum* DNA in the pGEM-3Zf(+) vector using a PCR generated 300 bp *sat* probe (primers used: aw1 CAGAC(C/G/T)CG(C/T)AA(C/T)CC(G/C)ATGCA and aw2 TC(G/A)CG/G/A)CC(G/C)A(C/T)(G/A) ATGAAGTG). The plasmid was digested with *Eco*RI, blunt-ended with Klenow polymerase and subsequently digested with *Sal*I. The resulting 2.6 kb fragment was cloned into pSUP202 [39] that had been cut with *Bam*HI, blunt-ended and then digested with *Sal*I. This led to plasmid pAW201 which was used to introduce the blunt-ended, 2.3-kb interposon from plasmid pHP45Ω [40] into a single *Nsi*I site residing in the center of the *sat* gene, resulting in plasmid pAWP202. This plasmid was transferred to *A. vinosum* SM50 [26] by conjugation from *E. coli* SM10 [39] according to [41]. The genotype of the resulting double-cross over mutants was verified by Southern hybridization.

### Cloning, Protein Expression and Purification

The *sat* gene from *A. vinosum* was amplified from chromosomal DNA by PCR using primers aw9 (5′-AGGAGGTTCGCATATGATCAAGCCAG9-3′ and aw10 (5′-GGTCAACTCAGGATCCTC TACACAAG99-3′) and subsequently cloned between the *Nde*I and *Bam*HI sites of expression vector pET-11a (Novagen, Darmstadt). The protein was overexpressed in *E. coli* BL21(DE3) pLysS cells (Novagen, Darmstadt) using LB medium at 303 K and 225 rpm agitation. Expression of ATPS was induced at an OD_600_ of 0.6–0.8 by adding IPTG to a final concentration of 0.5 mM. Cells were harvested 3 hours after induction by centrifugation and stored at −80°C until further use.

For kinetic characterization, His-tagged protein was purified by Nickel-chelate affinity chromatography essentially according to the manufacturer’s instructions. The column was washed with a buffer containing only 50 mM instead of 60 mM imidazole and ATPS was eluted by application of a linear imidazole gradient (70–180 mM) followed by dialysis against 50 mM Tris-HCl, pH 7.9. Final purification of the recombinant protein was achieved by gel filtration on Superdex 75 (GE Healthcare, Munich) using the same buffer (Fig. S3).

For crystallization ATPS was purified as follows: thawed cells were suspended in 20 mM Tris-HCl pH 8.0 and disrupted by sonification. Insoluble components were removed by centrifugation at 171500×*g* and 4°C. The supernatant was adjusted to an ammonium sulfate concentration of 0.75 M and loaded onto a Phenyl-Sepharose column (1.6 cm×10 cm; GE Healthcare, Muenchen). ATPS eluted in three peaks; fractions from the second and third peak were concentrated by ultrafiltration (Amicon Ultra-4, 10 kDa cutoff; Millipore, Eschborn) and dialyzed against 50 mM, Tris-HCl pH 8.0 (Membrane “Visking”, Roth, type 20/32). The protein solution was loaded onto a Resource Q column (0.5 cm×10 cm; GE Healthcare, Muenchen) and eluted by a linear gradient (0–1 M NaCl) at about 0.16 M NaCl. After concentration by ultracentrifugation purification was completed by using a Superdex 200 HR 10/30 gel filtration column (GE Healthcare, Muenchen) equilibrated with 50 mM Tris-HCl pH 8.0 and 150 mM NaCl. Protein was concentrated to 10 mg/ml and stored at 100 K in 50 mM Tris-HCl pH 8.0.

### Enzyme Assays

ATP sulfurylase was routinely measured in the thermodynamically favoured direction of ATP generation from APS and PP_i_ using the coupled spectrophotometric assay described by [4] with slight modifications. In a total volume of 1.0 ml the reaction mixtures contained 100 mM Tris-HCl, pH 8.0, 20 mM β-D-glucose, 4 mM MgCl_2_, 0.5 mM Na-NADP, 1 mM Na-PP_i_, 10 units glucose-6-phophate dehydrogenase, 7.5 units hexokinase, 0.2 mM APS and enzyme extract. Reduction of NADP was followed at 340 nm (ε = 6.22 mM^−1^cm^−1^) and 30°C.

The continuous spectrophotometric molybdolysis (AMP release) assay [42] was used for characterizing the activity of the enzyme on the “short-circuiting” inorganic substrate molybdate instead of sulfate. The molybdate- and ATP-sulfurylase-dependent formation of AMP was monitored in a reaction mixture (1 ml) containing the following final concentrations of reagents: 50 mM Tris-HCl, pH 8.0, 50 mM MgCl_2_, 4 mM phosphoenolpyruvate, 0.3 mM Na-NADH, 1 mM KCl, 50 mM NaMoO_4_, 10 mM ATP, 20 units pyruvate kinase, 22 units lactate dehydrogenase, 14 units adenylate kinase and enzyme extract. Oxidation of NADH was followed at 340 nm and 30°C.

Primary plots of initial rate against substrate concentration fit to the Michealis-Menten equation were created and analyzed by non-linear regression using Graph Pad Prism (version 6.0; Graph Pad).

Presence of APS reductase activity in *A. vinosum* wild type and mutant strains was assessed via thin-layer chromatography of APS reductase reactions as described in [26].

### Crystallization and X-ray Data Collection

Initial crystals were obtained at a temperature of 291 K within a vapor diffusion experiment using the crystallization kits Classic from Jena Bioscience and MDL I+II of Molecular Dimensions. Optimization led to a drop content of 2 µl enzyme solution (10 mg ml^−1^) in a buffer consisting of 50 mM Tris-HCl pH 8.0 and 2 µl reservoir solution (1.5 M potassium sodium tartrate and 100 mM MES pH 6.5). Crystals grew in the space group P2_1_ with unit cell parameters of a = 73.3 Å, b = 97.0 Å, c = 73.5 Å and β = 117.6° and two subunits in the asymmetric unit (V_M_ = 2.4 Å^3^Da^−1^, solvent content 49.2%). For cryoprotection, crystals were placed for several minutes into a buffer containing 1.5 M potassium sodium tartrate, MES pH 6.5 and 15% glycerol. Data were collected at 100 K up to a resolution of 1.6 Å at the ESRF in Grenoble, France and processed with the HKL program suite (Table 1) [43]. Soaking and co-cystallization attempts with APS, ATP or ATP analogues and MgCl_2_ failed.

### Structure Determination, Refinement and Substrate Modeling

The structure was solved by the molecular replacement, using the structure of ATPS from “*Candidatus* Endoriftia persephone” (PDB code 1 JHD; [30]) as a search model. A cross-rotational search followed by a translational search was performed utilizing the program EPMR [44]. Model building was performed using O [45] and refinement with CNS [46] and REFMAC5 [47] within the CCP4 program suite [48]. 5% of the data were randomly set aside as test data for the calculation of R*_free_* [49]. Refinement parameters and the PDB accession code are included in Table 1. Figures were generated using PyMol (Schrodinger, LLC). Docking simulations have been performed using AutoDock [34] on the basis of the rigid protein model as a search template.

## Supporting Information

Figure S1 **Simulation of the proposed ATPS-APS-PP_i_ adduct (A) and ATPS-ATP product (B).** The highly conserved ^199^QXRNXXHXXH^208^ motif is involved in the coordination of the sulfate and phosphate moiety.
(PDF)

Figure S2 **Molecular surface representation with emphasis on the deep cleft within the catalytic domain.** The substrate binding pocket is designed to perfectly position APS and PP_i_ in adequate proximity. The calculation of the electrostatic potential of the surface using the program APBS (Baker, N. A., Sept. D., et al. (2001). “Electrostatics of nanosystems: application to microtubules and the ribosome.” Proc Natl Acad Sci U S A 98∶10037-41.) revealed an overall negative potential on the surface (red) and around the substrate binding pocket a positive charge (blue).
(PDF)

Figure S3 **SDS–PAGE (10%) of recombinant** ***A. vinosum*** **ATP sulfurylase.** The gel was stained with Coomassie brilliant blue. Protein purity was assessed after Nickel-chelate affinity chromatography (lane 1) and subsequent gel filtration chromatography (lane 2). Protein loaded: lane 1, 2 µg; lane 2, 1 µg.
(PDF)
