# Correction: Structural, Biochemical and Genetic Characterization of Dissimilatory ATP Sulfurylase from *Allochromatium vinosum*

**DOI:** 10.1371/annotation/fab66ad6-bdfa-4f76-9c39-08f28a92494d

**Published:** 2013-10-01

**Authors:** Kristian Parey, Ulrike Demmer, Eberhard Warkentin, Astrid Wynen, Ulrich Ermler, Christiane Dahl

In the author byline, the corresponding authors should be Ulrich Ermler and Christiane Dahl, who can be reached, respectively, at ulrich.ermler@mpibp-frankfurt.mpg.de and ChDahl@uni-bonn.de

Astrid Wynen should be noted as having the current address: Envita Pharm GmbH, Industriestr. 29, Zuzwil, Switzerland

