# Supplementary material for: Structural, Biochemical and Genetic Characterization of Dissimilatory ATP Sulfurylase from Allochromatium vinosum
Source: PLoS One. 2013 Sep 20;8(9):e74707. doi: 10.1371/journal.pone.0074707 (PMC3779200; doi:10.1371/journal.pone.0074707)
Supplement: Figure S1 — Simulation of the proposed ATPS-APS-PPi adduct (A) and ATPS-ATP product (B). The highly conserved 199QXRNXXHXXH208 motif is involved in the coordination of the sulfate and phosphate moiety. (PDF) [file pone.0074707.s001.pdf]

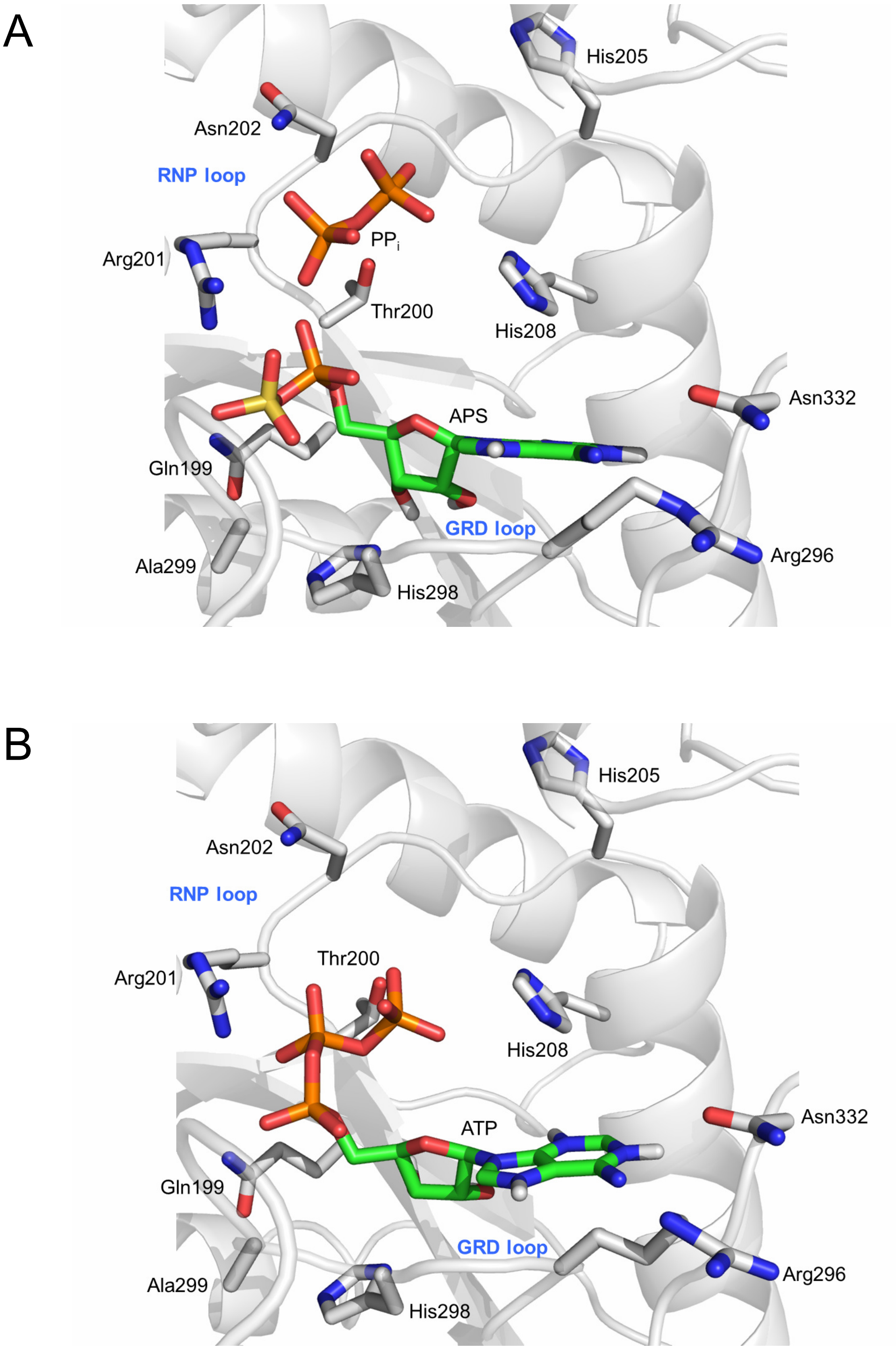

Fig. S1: Simulation of the proposed ATPS-APS-PP_i adduct (A) and ATPS-ATP product (B). The highly conserved ^{199}QXRNXXHXXH^{208} motif is involved in the coordination of the sulfate and phosphate moiety.
