# Supplementary material for: Structural, Biochemical and Genetic Characterization of Dissimilatory ATP Sulfurylase from Allochromatium vinosum
Source: PLoS One. 2013 Sep 20;8(9):e74707. doi: 10.1371/journal.pone.0074707 (PMC3779200; doi:10.1371/journal.pone.0074707)
Supplement: Figure S2 — Molecular surface representation with emphasis on the deep cleft within the catalytic domain. The substrate binding pocket is designed to perfectly position APS and PPi in adequate proximity. The calculation of the electrostatic potential of the surface using the program APBS (Baker, N. A., Sept. D., et al. (2001). “Electrostatics of nanosystems: application to microtubules and the ribosome.” Proc Natl Acad Sci U S A 98∶10037-41.) revealed an overall negative potential on the surface (red) and around the substrate binding pocket a positive charge (blue). (PDF) [file pone.0074707.s002.pdf]

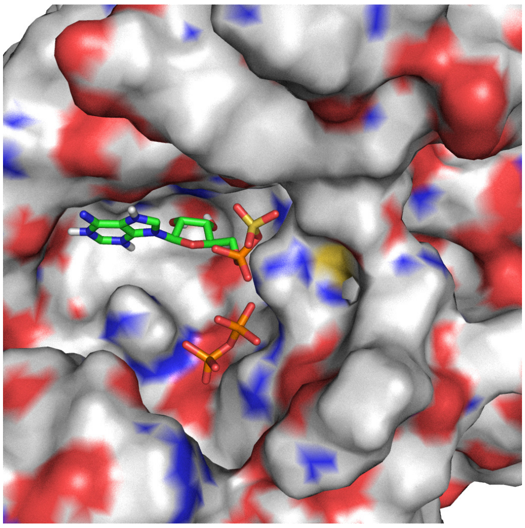

Fig. S2: Molecular surface representation with emphasis on the deep cleft within the catalytic domain. The substrate binding pocket is designed to perfectly position APS and PP_i in adequate proximity. The calculation of the electrostatic potential of the surface using the program APBS (Baker, N. A., Sept. D. et al. (2001). "Electrostatics of nanosystems: application to microtubules and the ribosome." Proc Natl Acad Sci USA 98: 10037-41.) revealed an overall negative potential on the surface (red) and around the substrate binding pocket a positive charge (blue).
