# Supplementary material for: Structural, Biochemical and Genetic Characterization of Dissimilatory ATP Sulfurylase from Allochromatium vinosum
Source: PLoS One. 2013 Sep 20;8(9):e74707. doi: 10.1371/journal.pone.0074707 (PMC3779200; doi:10.1371/journal.pone.0074707)
Supplement: Figure S3 — SDS–PAGE (10%) of recombinant A. vinosum ATP sulfurylase. The gel was stained with Coomassie brilliant blue. Protein purity was assessed after Nickel-chelate affinity chromatography (lane 1) and subsequent gel filtration chromatography (lane 2). Protein loaded: lane 1, 2 µg; lane 2, 1 µg. (PDF) [file pone.0074707.s003.pdf]

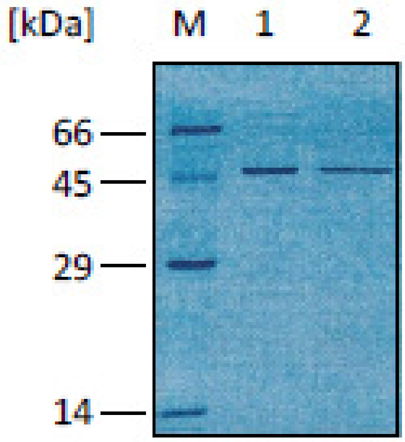

Fig. S3. SDS–PAGE (10%) of recombinant *A. vinosum* ATP sulfurylase. The gel was stained with Coomassie brilliant blue. Protein purity was assessed after Nickel-chelate affinity chromatography (lane 1) and subsequent gel filtration chromatography (lane 2). Protein loaded: lane 1, 2 µg; lane 2, 1 µg.
